# Professional Habits

**Published:** 1895-06

**Authors:** 


PROFESSIONAL HABITS.—In the May number of *Blackwood* Dr, Louis Robinson treats of the mode in which external circumstances (especially professional habits) tend to mold human countenances into facial expressions which, very frequently at least, are misleading as to the chief significance of what they convey to us. For example, the tendency of a compressed lip is to suggest strength of will, whereas what it does really express is the habit of struggling against inclinations and temptations which are apt to be too strong for us; in other words, it tells, he thinks, rather of weakness than of strength, though of weakness which is training itself to be strong. According to Dr. Robinson, expression tells you not so much what a man is as what he tries to be; and as he tries to be what he is not, we constantly find him wearing a mask which conceals his weakness, through it reveals his sins.

Our countenances are marks on which the habitual attitude of the character is mirrored; but the habitual attitude of the character is not an index of the greatest strength of the character, but sometimes at least of its greatest weakness. It may tell you whether a man is "rowing hard against the stream" or rowing feebly against it, but it does not tell you where the stream would bear him if he did not row at all.

Dr. Robinson holds that as the artist, as an artist, succeeds by giving his mind up to the particular mood or feeling which he is most anxious to delineate, he very often disguises his true self by a false appearance of self indulgence. He regards the true actor's face as almost necessarily a mask, since it takes on the form of so many different emotions in turn that they counteract each other and leave a kind of neutral expression, such as extinguishes all traces of the habitual bias of the character itself.

Nothing is commoner than a complete misnonstruction of the meaning of certain countenances. Some which appear to bear their amiable drift written plainly in a genial and kindly smile, are really expressions of nothing but empty good-nature, without a vestige of thoroughgoing willingness to serve others which appears to be promised by their superficial sunshine. Other faces which are clouded and melancholy, and appear to convey the expression of covert purposes, are really expressive of high and tenacious ideas, which have never had their full satisfaction in practical life, and crowned with far less success, than those of the minds whose frank and genial surface covers a shallow and commonplace nature.

Though a merely professional blandness may excite a great deal more distrust than it ought to excite to the ear of an unpracticed listener, yet with a very little training it will learn to distinguish even in the "bland physician's" courtly voice the accent of genuineness which is more than half merged in that of persuasive and sympathetic warning. Faces are often effectual, and what is more, perfectly unintentional masks—where voices are the plainest tell tales.

